# Supplementary material for: The Curse of the Red Pearl: A Fibroblast-Specific Pearl-Necklace Mitochondrial Phenotype Caused by Phototoxicity
Source: Biomolecules. 2025 Feb 19;15(2):304. doi: 10.3390/biom15020304 (PMC11853634; doi:10.3390/biom15020304)
Supplement: Supplementary file 1 [file biomolecules-15-00304-s001.zip › biomolecules-3416892-supplementary.pdf]

Supplemental information

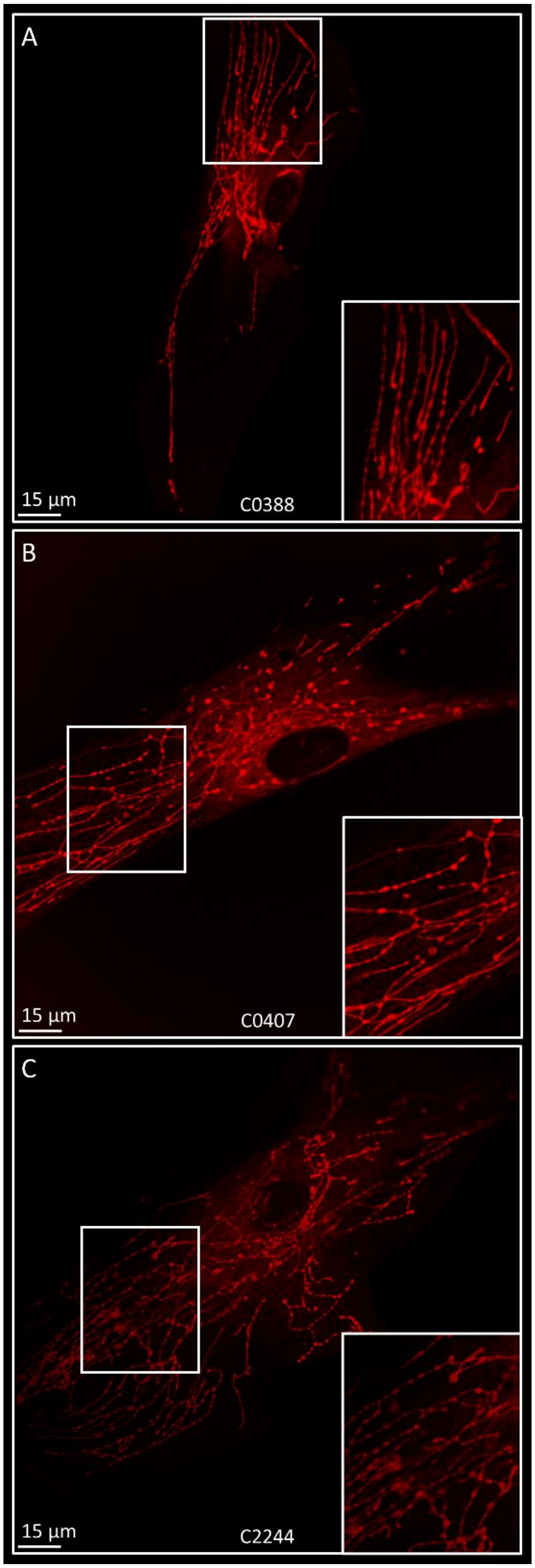

Figure S1: The pearl-necklace phenotype is present in multiple dermal fibroblast cell lines. A) c0388 cells. B) c0407 cells. C) c2244 cells.

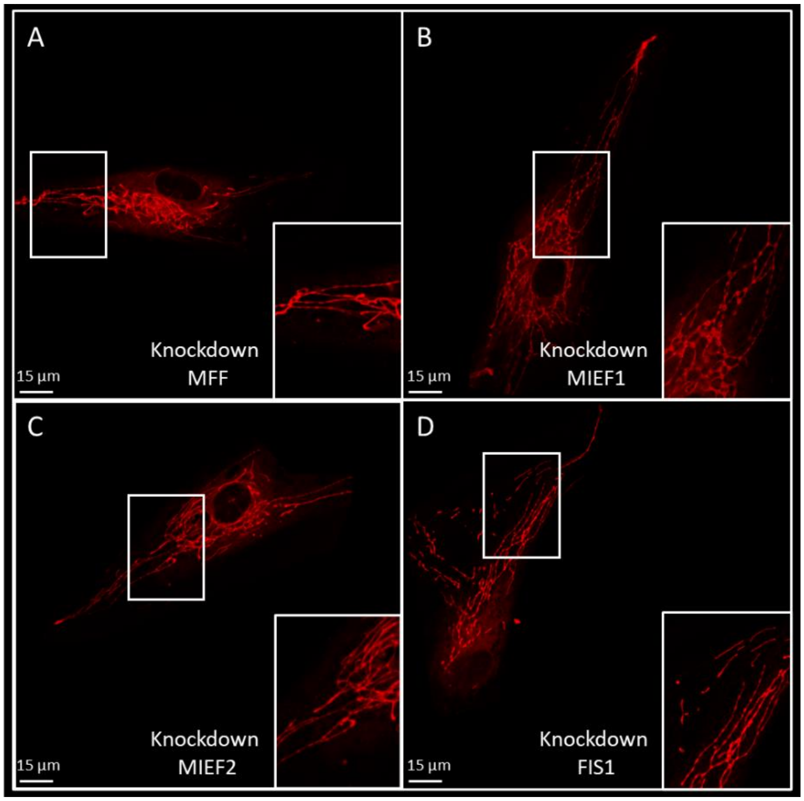

Figure S2: The pearl-necklace phenotype is present upon knockdown of fission factors. A) MFF knockdown. B) MIEF1 knockdown. C) MIEF2 knockdown. D) FIS1 knockdown.

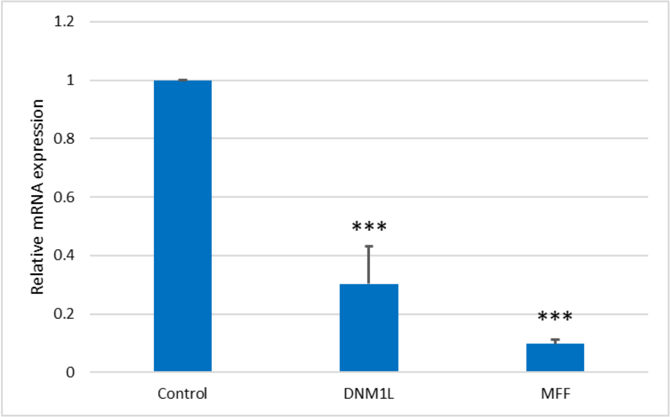

*Figure S3: Knockdown efficiency of DNM1L (Drp1) and MFF knockdown in normal human dermal fibroblasts. Both knockdown conditions show at a decrease in mRNA expression. ***p < 0.001 (Independent sample T-test, significance level adjusted for multiple testing).*

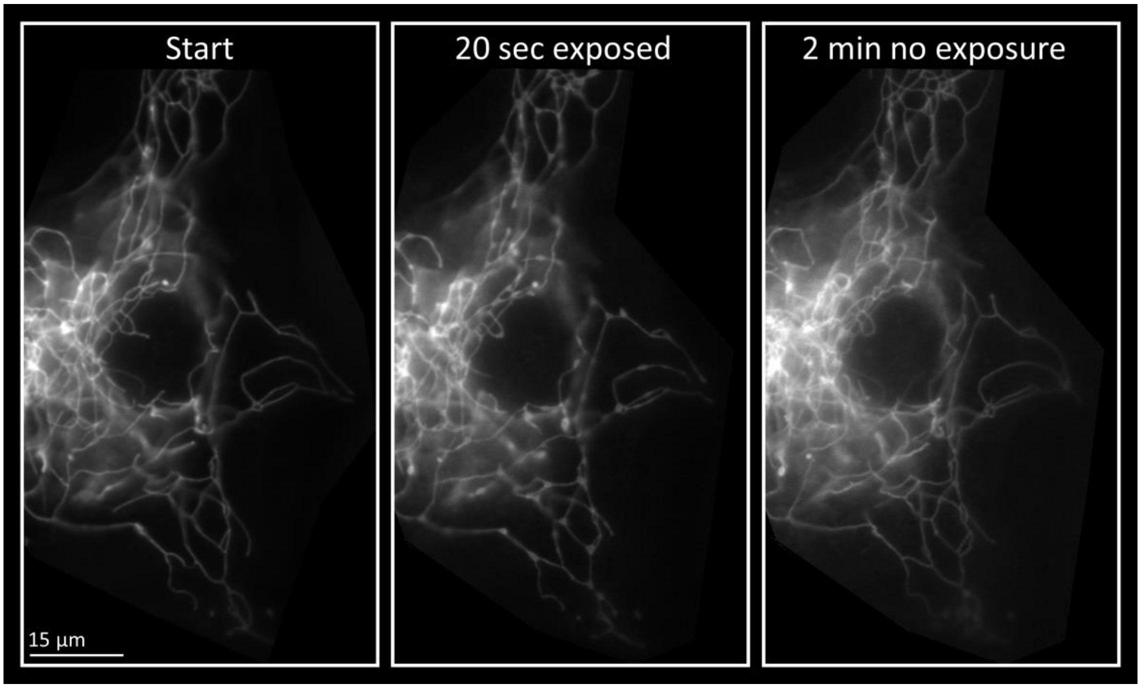

Figure S4: Following formation of the pearl-phenotype, the mitochondrial morphology can be reversed by discontinuation of the laser excitation.

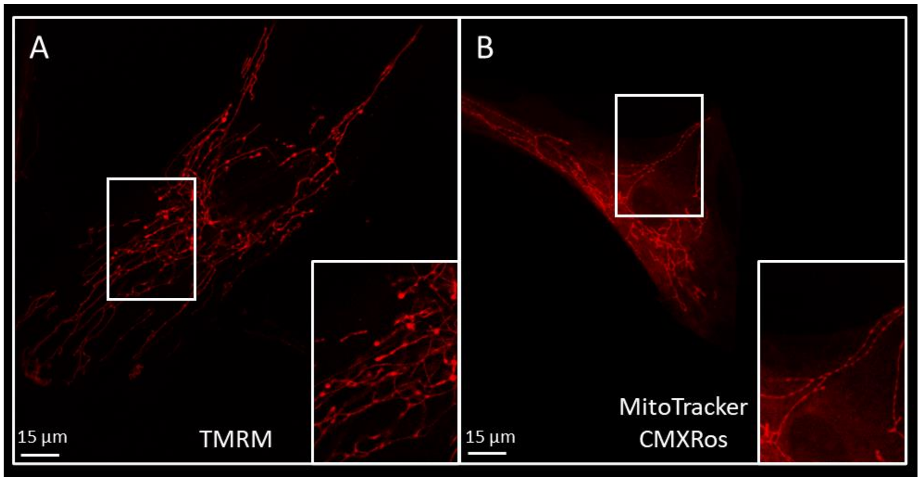

Figure S5: The pearl-necklace phenotype is present when mitochondria are stained with different red live cell stainings. A) TMRM. B) MitoTracker CMX-ROS.
